# Structure-Based Identification of Potent Natural Product Chemotypes as Cannabinoid Receptor 1 Inverse Agonists

**DOI:** 10.3390/molecules23102630

**Published:** 2018-10-13

**Authors:** Pankaj Pandey, Kuldeep K. Roy, Haining Liu, Guoyi Ma, Sara Pettaway, Walid F. Alsharif, Rama S. Gadepalli, John M. Rimoldi, Christopher R. McCurdy, Stephen J. Cutler, Robert J. Doerksen

**Affiliations:** 1 Department of BioMolecular Sciences, Division of Medicinal Chemistry, School of Pharmacy, The University of Mississippi, MS 38677, USA; ppandey@olemiss.edu (P.P.); kuldeepkroy@gmail.com (K.K.R.); hainingliu14@gmail.com (H.L.); gyma@olemiss.edu (G.M.); pettaway@email.unc.edu (S.P.); alsharif@med.wayne.edu (W.F.A.); rama@olemiss.edu (R.S.G.); jrimoldi@olemiss.edu (J.M.R.); cmccurdy@cop.ufl.edu (C.R.M.); sjcutler@cop.sc.edu (S.J.C.); 2 National Institute of Pharmaceutical Education and Research, 168, Manicktala Main Road, Kolkata 700054, WB, India; 3 Research Institute of Pharmaceutical Sciences, School of Pharmacy, University of Mississippi, MS 38677, USA

**Keywords:** structure-based virtual screening, cannabinoid receptors, docking, virtual screening, radioligand binding assay

## Abstract

Natural products are an abundant source of potential drugs, and their diversity makes them a rich and viable prospective source of bioactive cannabinoid ligands. Cannabinoid receptor 1 (CB1) antagonists are clinically established and well documented as potential therapeutics for treating obesity, obesity-related cardiometabolic disorders, pain, and drug/substance abuse, but their associated CNS-mediated adverse effects hinder the development of potential new drugs and no such drug is currently on the market. This limitation amplifies the need for new agents with reduced or no CNS-mediated side effects. We are interested in the discovery of new natural product chemotypes as CB1 antagonists, which may serve as good starting points for further optimization towards the development of CB1 therapeutics. In search of new chemotypes as CB1 antagonists, we screened the in silico purchasable natural products subset of the ZINC12 database against our reported CB1 receptor model using the structure-based virtual screening (SBVS) approach. A total of 18 out of 192 top-scoring virtual hits, selected based on structural diversity and key protein–ligand interactions, were purchased and subjected to in vitro screening in competitive radioligand binding assays. The in vitro screening yielded seven compounds exhibiting >50% displacement at 10 μM concentration, and further binding affinity (K_i_ and IC_50_) and functional data revealed compound **16** as a potent and selective CB1 inverse agonist (K_i_ = 121 nM and EC_50_ = 128 nM) while three other compounds—**2**, **12**, and **18**—were potent but nonselective CB1 ligands with low micromolar binding affinity (K_i_). In order to explore the structure–activity relationship for compound **16**, we further purchased compounds with >80% similarity to compound **16**, screened them for CB1 and CB2 activities, and found two potent compounds with sub-micromolar activities. Most importantly, these bioactive compounds represent structurally new natural product chemotypes in the area of cannabinoid research and could be considered for further structural optimization as CB1 ligands.

## 1. Introduction

G-protein coupled receptors (GPCRs) represent the largest single group of targets for approximately 40% of FDA-approved drugs [1,2]. Functional selectivity among these targets is highly recommended for successful drug discovery. The cannabinoid (CB) receptors belong to the Class A, membrane-bound rhodopsin-like family of GPCRs characterized by seven transmembrane (TM) helices/domains [3,4]. One of the two CB receptor subtypes, CB1, is especially abundant in the central nervous system [5,6], but it is also found in peripheral tissues [7]. The other subtype, CB2 [8], is mostly present in the immune system [9] and the spleen [10], though evidence suggests that the CB2 receptor is also expressed in the CNS in small proportions [11,12,13]. CB1 receptor antagonists are clinically established to be effective in treating obesity [14], obesity-related cardiometabolic disorders [15], and substance abuse [16,17]. The druggability of CB1 receptor antagonists has been clinically validated through the drug rimonabant (SR141716A) [18], which showed progressive and prolonged weight loss in overweight patients [19] and improvements in associated metabolic disorders [20,21] in phase III clinical trials. Unfortunately, due to undesirable CNS side effects associated with rimonabant such as depression, anxiety, nausea, and dizziness, prescription of rimonabant was halted in Europe and was never authorized in the USA [22]. A recent strategy was applied to overcome or minimize CNS-mediated side effects associated with CB1 inverse agonists by developing compounds that do not cross the blood–brain barrier and that act only on peripheral tissues. This approach is being used by several researchers who have shown that peripherally restricted CB1 antagonists and inverse agonists reduce body weight and improve metabolic profile in vivo, while being devoid of CNS-mediated side effects [23,24]. On the other hand, the CB2 receptor is also an important target for the discovery of therapeutics for neuro-inflammation, cardiometabolic disorder, cardiac ischemia, renal ischemia–reperfusion injury, and other diseases/disorders [25,26,27,28,29].

A proper understanding of the cannabinoid receptors, their potential binding pockets, and CB–ligand binding modes can assist the discovery of new potential therapeutics targeting CB receptors. In fall 2016, two inactive-state CB1 X-ray crystal structures [30,31] were published, and in spring 2017, two active-state CB1 X-ray crystal structures [32] were published. In many cases, molecular modeling tools have been useful in understanding the putative 3D structures of CB receptors, modes of protein–ligand interactions, and important physicochemical and structural requirements for effective CB modulation [33,34]. In the search for novel molecules targeting the endocannabinoid system, past research efforts were devoted towards developing promising analogs or isosteres of rimonabant [35,36,37] that are active against the CB1 receptor, but none of the discovered compounds reached phase III clinical trials. Several studies have been reported that showed the successful identification of drug-like sub-micro/nanomolar CB1 antagonists using bovine rhodopsin-based homology models [38,39,40] and pharmacophore modeling [41,42,43].

Natural products continue to be a major source of new and structurally diverse lead compounds. Natural product drug discovery outlines many important drugs that revolutionized the treatment of several diseases over several decades [44,45,46]. In view of this, we embarked on a journey to discover new natural product chemotypes as CB1 inverse agonists/antagonists. We utilized a protein structure-based virtual screening (SBVS) approach starting from the all-purchasable ZINC12 natural products subset [47], which has led to identification of novel chemotypes as CB1 inverse agonists as described in this paper.

## 2. Results and Discussion

### 2.1. Structure-Based Virtual Screening of the ZINC12 Subset of Natural Products

**Figure 1:**
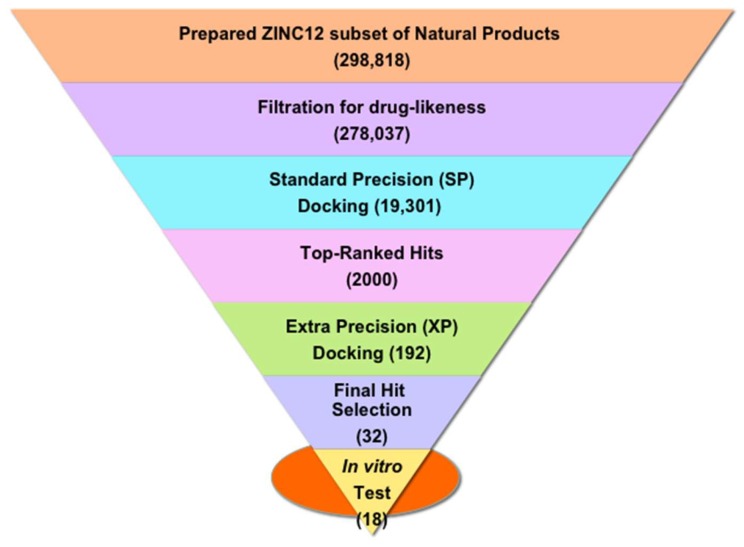
The workflow used for protein structure-based virtual screening in this study. The number of compounds obtained at each step of virtual screening is shown in parentheses.

We previously reported an antagonist-bound CB1 homology model based on the bovine rhodopsin template structure [48]. The best CB1 model was identified through an enrichment study considering a set of known active and inactive CB1 antagonists. This model was understood to represent the inactive state of the CB1 receptor, following various characteristics of the inactive GPCR state identified through X-ray crystallography [49]. Aiming towards the identification of novel natural product chemotypes targeting the CB1 receptor, we screened in silico the ZINC12 subset of 278,037 commercially available, drug-like natural products for their potential binding with our CB1 receptor model. Figure 1 depicts the schematic virtual screening workflow used in the present study. We docked the prepared set of ~0.3 million drug-like compounds into the antagonist-bound CB1 model using the Glide [50] module of the Schrödinger suite (www.schrödinger.com) considering its standard precision (SP) method that in return yielded a total of 19,301 compounds ranked based on the SP-GlideScore. We considered a set of 2000 top-scoring compounds (SP-GlideScore cutoff ≤ −9.0 kcal/mol) and these compounds were subjected to another stage of Glide docking utilizing its more precise and robust extra-precision (XP) method [51]. This stage afforded a total of 618 successfully docked compounds ranked based on the XP-GlideScore. We selected a total of 192 compounds considering an XP-GlideScore cutoff of ≤ −8.0 kcal/mol. In order to select structurally diverse chemotypes, we clustered the 192 compounds based on docking score and fingerprint properties, and the final selection was made through visual inspection of predicted binding modes for polar interaction with the key residue Lys192^3.28^ along with other polar and hydrophobic interactions. The clustering returned a final list of 32 compounds, from which a set of 18 structurally diverse compounds (Figure S1) were purchased on the basis of their cost and immediate availability, and subjected to in vitro screening for CB receptor activity.

### 2.2. In Vitro Screening in the Competitive Radioligand Binding Assay

In the preliminary screening, all 18 purchased compounds were subjected to in vitro CB1 and CB2 activity assays at a single concentration of 10 µM. The chemical structures are shown in Figure S1, and the observed percentage displacement (%) of radioligand at the CB receptors along with docking scores and other drug-like properties of these 18 compounds are summarized in Table S1 in the Supporting Information. As shown in Figure S1, these compounds are structurally unique from any reported small-molecule CB ligands, confirming the chemotype novelty of all the tested compounds. The highly potent and nonselective CB agonist CP55,940, a nonclassical bicyclic cannabinoid, was used as a radioligand in the competitive radioligand binding assay because it mimics the therapeutic effect of the naturally occurring compound Δ^9^-THC, and it binds in the same orthosteric active site where known CB agonists bind. In addition, it is an extensively studied radioligand frequently used in the literature for the cannabinoid assays [52,53].

**Figure 2:**
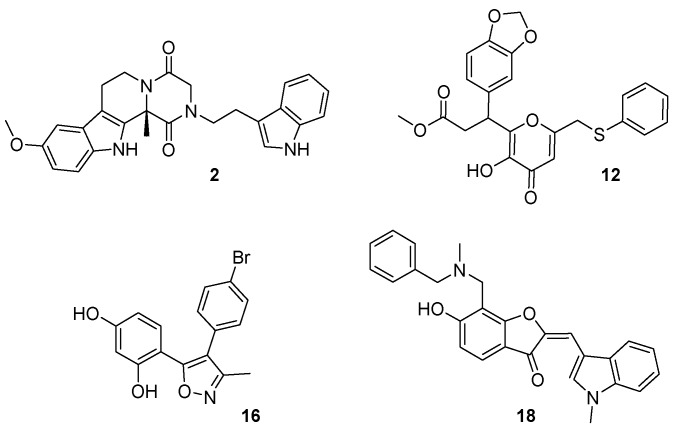
Chemical structures of the four molecules newly identified as cannabinoid (CB) ligands.

**Figure 3:**
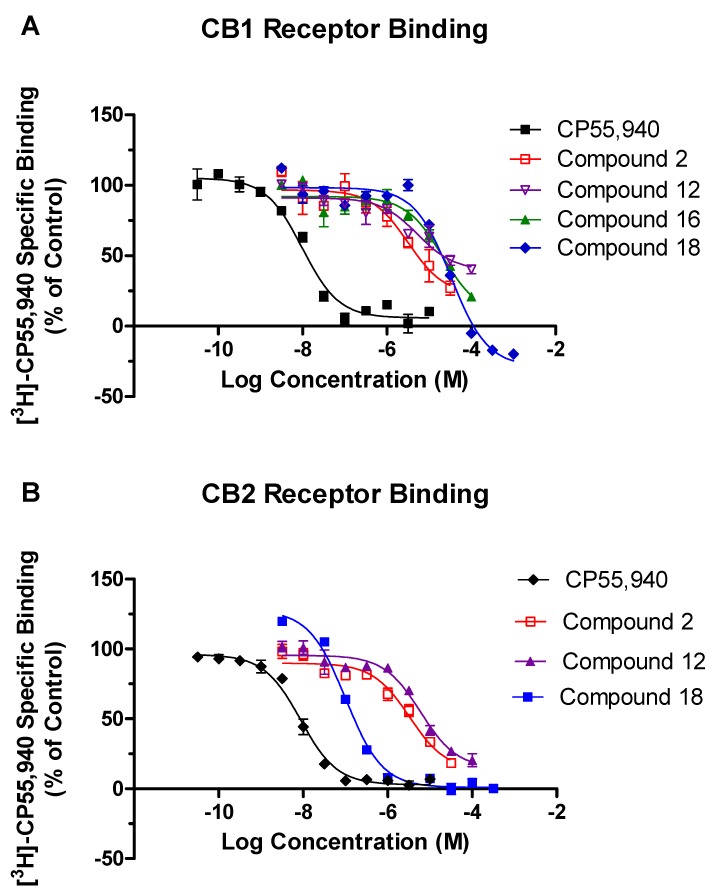
The binding displacement curves obtained for compounds **2**, **12**, **16**, and **18** for the CB1 receptor (**A**) and CB2 receptor (**B**) in the cannabinoid radioligand binding assay. CP55,940 was used as a positive control.

From among the 18 compounds evaluated in the competitive radioligand binding assay (Table S1), 4 structurally distinct compounds (Figure 2) showed significant displacement at the CB1 receptor yielding binding affinities K_i_ ≤ 12.3 µM (Figure 3A and Table 1). Meanwhile, three of these compounds also exhibited low micromolar CB2 displacement with K_i_ ≤ 3.0 µM (Figure 3B and Table 1). Two of the three most promising compounds, **2** (CB1 K_i_ = 1.6 µM; CB2 K_i_ = 1.6 µM) and **12** (CB1 K_i_ = 2.6 µM; CB2 K_i_ = 3.0 µM), were observed to be nonselective CB ligands with high binding affinity towards both of the CB receptor subtypes. Broadly, these newly identified cannabinoid ligands represent four chemical classes, namely, hexahydropyrazinone (Compound **2**), pyran (Compound **12**), isoxazole (Compound **16**), and benzofuran (Compound **18**), and are appropriate starting points for further optimization of their CB affinity and selectivity.

Among the 18 screened compounds, 8 were tested in racemic form (Figure S1, Table S1). The particular stereoisomers of these eight compounds which had the better predicted docking scores were either unavailable or too costly. That they were tested in racemic form may be one of the reasons why these compounds did not show binding affinity as good as that predicted for the CB1 receptor. One of the racemic compounds **3**, showed >50% displacement at the CB2 receptor (~10% CB1 displacement), while another racemic compound **12**, showed >50% displacement of radioligand at both CB1 and CB2 receptors at 10 µM (Table S1). Compound **3** displayed an acceptable binding affinity for the CB2 receptor with a K_i_ value of 2.6 µM.

Further binding analysis for the three compounds **7**, **15**, and **17**, exhibiting ≥50% displacement at the CB1 receptor when tested at 10 μM, revealed that the binding curves of the compounds plateaued well before reaching 100% receptor occupancy, indicating no true affinity for the CB1 receptor (Figure S2). When full curves could not be obtained due to low affinity or solubility limits of the compounds, the values are reported as greater than the last tested concentration (Table 1). Compound **18** (which was yellow in solution) has a conjugated π-bond system, which may be considered as a chromophore and whose presence is responsible for the color of the compound. At higher concentrations, a chromophore can interfere with the radioligand detection system due to color quenching whereby some fraction of the photons produced by the MicroScint^TM^-20 cocktail are absorbed by the colored compound before reaching the radioligand detector photomultiplier tube, which causes significant changes in the radionuclide spectrum and loss of count rate [TopCount Topics #15, “Quench and Quench Correction”, Packard Instrument Company]. Therefore, it was difficult to precisely determine if the lower rate of radioligand detection was because the radioligand was bound to the receptor or because the chromophore was interfering with the radioligand detection system. Thus, the IC_50_ and K_i_ values for such compounds possessing chromophore systems could not be accurately determined using our current assay system.

### 2.3. In Vitro GTPγS Functional Assays for CB1 and CB2 Receptors

**Figure 4:**
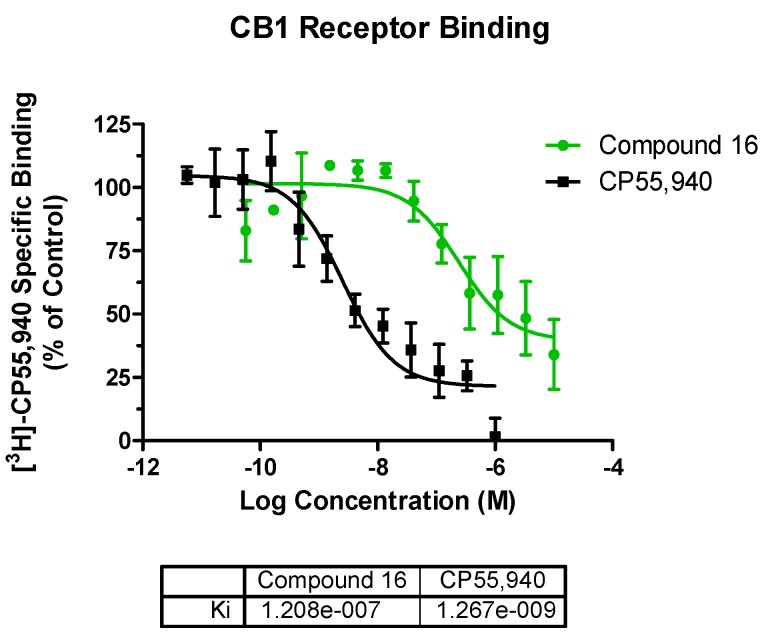
The binding displacement curves obtained when **16** was rescreened in the cannabinoid receptor 1 radioligand binding assay. CP55,940 was used as a positive control.

**Figure 5:**
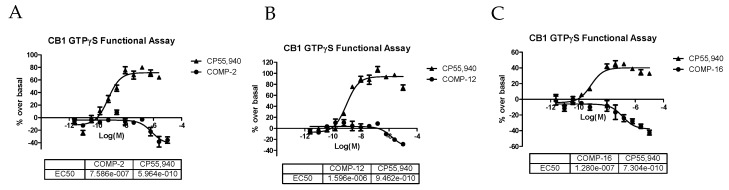
GTPγS functional curves for the CB1 receptor of compounds **2** (**A**), **12** (**B**), and **16** (**C**).

The GTPγS functional assay procedure is very similar to the binding assay methods and is used to analyze the functional effects of agonists, antagonists, and inverse agonists at GPCRs. We tested three compounds—**2**, **12**, and **16**—in the CB1 functional assay, and they were all determined to act as inverse agonists (Figure 5) with the most potent being **16** (EC_50_ = 128 nM). After seeing this result, we rescreened **16** in the competitive radioligand displacement assay (Figure 4) and obtained K_i_ = 121 nM (cf. Table 1). 

### 2.4. Protein–Ligand Interactions

**Figure 6:**
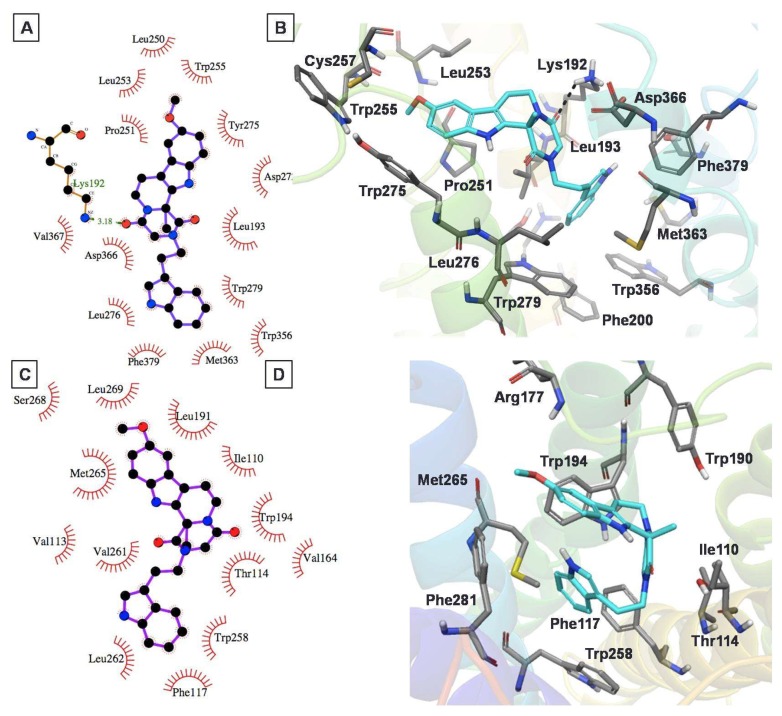
The putative binding mode for compound **2** into the CB1 (**A**,**B**) and CB2 (**C**,**D**) receptor models. Two-dimensional interaction views are shown on the left, while three-dimensional interaction views are shown on the right (ligand (cyan colored carbons) and protein binding site residues (dark grey colored carbons) are shown as sticks). The nonpolar hydrogens are not shown, for clarity.

Compound **2** (Figure 6A,B) exhibited a favorable binding mode with the CB1 receptor model including a key H-bond interaction with Lys192^3.28^ and a π–π stacking interaction with Trp279^5.43^. The methoxy-indole moiety also exhibited a π–π stacking interaction with another aromatic residue, Trp275^5.39^. The orientation of the indole ring inside the hydrophobic region of the CB1 receptor provided an added stabilization to the ligand through strong hydrophobic interactions. In contrast, docking analysis of this compound with the CB2 model (Figure 6C,D) showed that it exhibited π–π stacking interactions with an important residue, Trp194^5.43^, located on the extracellular side. Meanwhile, this compound also exhibited strong hydrophobic interactions with Trp258^6.48^, Phe117^3.36^, Val261^6.51^, and Leu262^6.52^ residues. Distance measurements revealed that the two polar residues, Arg177 and Thr114^3.33^, were within a distance of 3 Å of the methoxy and keto moieties of compound **2**, thus suggesting the possibility of H-bond formation, considering the flexibility of the protein binding pocket.

**Figure 7:**
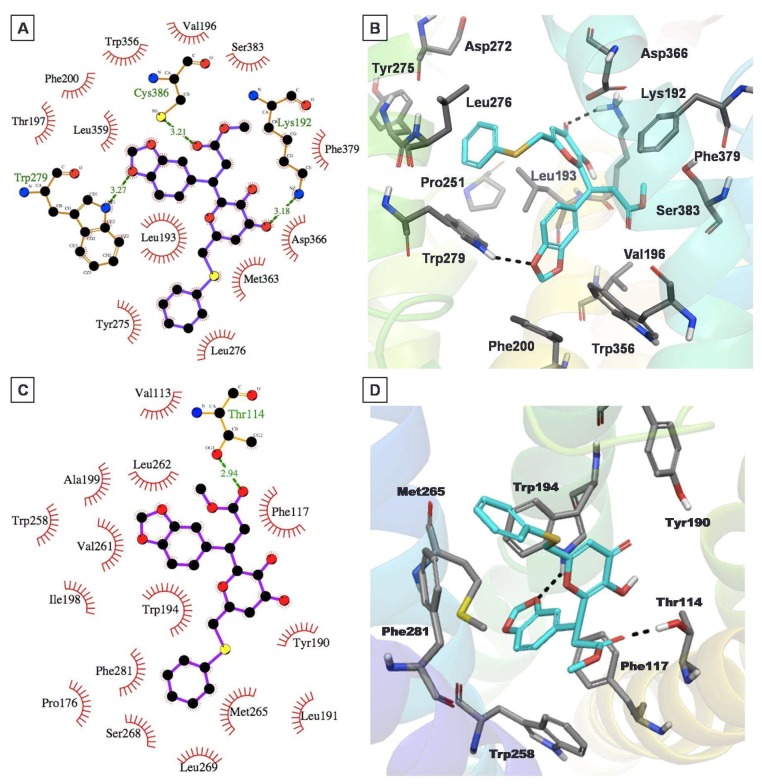
The putative binding mode for compound **12** into the CB1 (**A**,**B**) and CB2 (**C,D**) receptor models. Two-dimensional interaction views are shown on the left, while three-dimensional interaction views are shown on the right (ligand (cyan colored carbons) and protein binding site residues (dark grey colored carbons) are shown as sticks). The nonpolar hydrogens are not shown, for clarity.

The binding mode of compound **12** within the CB1 model demonstrated an H-bond interaction with key residue Lys192^3.28^ of the CB1 model (Figure 7A,B). The Trp279^5.43^ residue of CB1 exhibited dual interactions with this compound. The benzodioxazole oxygen of **12** participated in H-bond formation with NH of Trp279^5.43^, while the benzene ring showed π–π stacking interactions with Trp279^5.43^. Overall, this compound exhibited strong interactions with the CB1 receptor, helping to explain its good CB1 inhibitory activity, in the micromolar range (K_i_ = 2.6 µM). Meanwhile, it also exhibited quantitatively similar binding affinity for the CB2 receptor (K_i_ = 2.9 µM). Docking analysis of the *S* enantiomer of this compound to the CB2 model (Figure 7C,D) exhibited that the 1,3-benzodioxazole moiety had π–π stacking and H-bond interactions with the Trp194^5.43^ residue. In addition, its thiophenyl moiety exhibited π–π stacking interactions with Phe281^7.35^. Meanwhile, the 1,3-benzodioxazole and thiophenyl substructures were stabilized through strong hydrophobic interactions with several hydrophobic residues, namely Met265^6.55^, Pro176, Leu191^5.40^, Trp258^6.48^, Phe117^3.36^, Val261^6.51^, Leu262^6.52^, and Ile298^7.52^.

**Figure 8:**
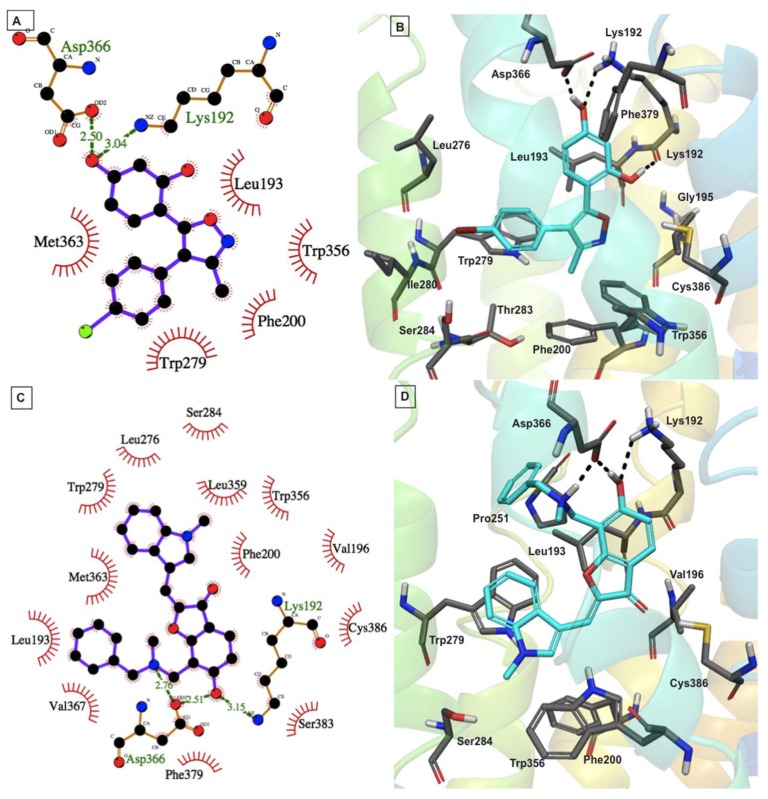
Putative binding mode of compounds **16** (**A**,**B**) and **18** (**C**,**D**) with the CB1 model. Two-dimensional interaction views are shown on the left, while three-dimensional interaction views are shown on the right (ligand (cyan colored carbons) and protein binding site residues (dark grey colored carbons) are shown as sticks). The nonpolar hydrogens are not shown, for clarity.

Compound **16** is smaller compared with the other three compounds (Figure 2). This compound fit well within the CB1 binding site and formed favorable binding interactions that included H-bond interactions with Lys192^3.28^ and Asp366^6.58^ and π–π stacking interactions of its oxazole and bromobenzene moieties with Trp356^6.48^ and Trp279^5.43^, respectively (Figure 8A,B).

Compound **18** showed stronger interactions with the CB1 model and thus was predicted to show better affinity toward CB1 in comparison to compound **16** (Figure 8C,D). Docking analysis revealed that compound **18** exhibited multiple H-bond interactions with Lys192^3.28^ and Asp366^6.58^, and an aromatic π–π stacking interaction with key residues Trp279^5.43^ and Trp379^7.35^ of the CB1 model. Unfortunately, the experimental activity of compound **18** could not be determined accurately by our current CB1 and CB2 assay system because of the presence of a chromophore group (conjugated double bond) in the ligand.

### 2.5. Analog Exploration

**Figure 9:**
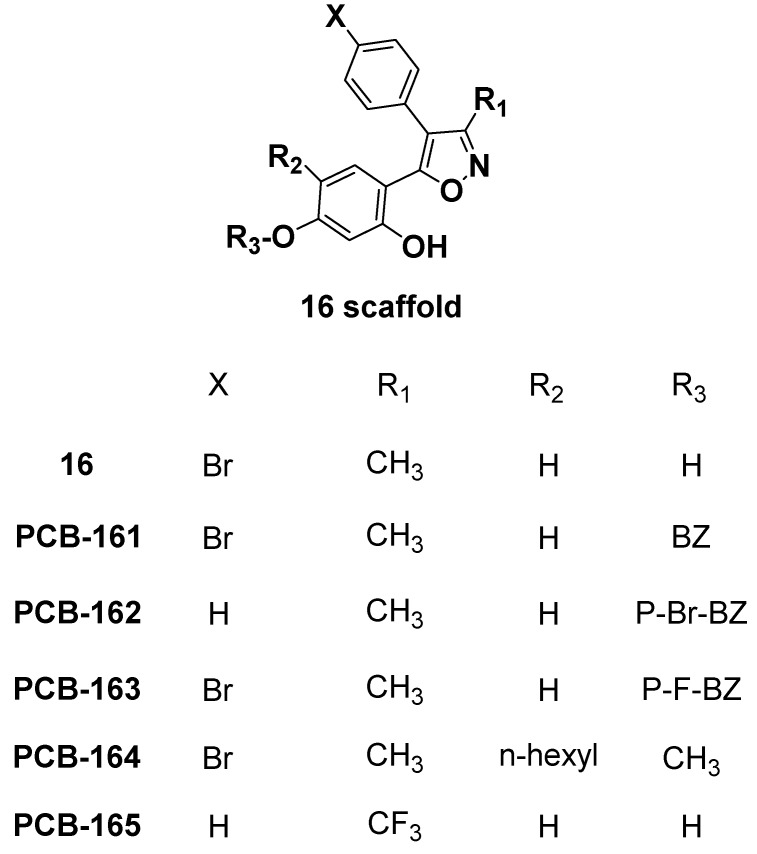
The analogs of compound **16**.

The functional CB1 GTPγS assays revealed that virtual screening hit compound **16** is a CB1 inverse agonist with a potent functional EC_50_ of 128 nM. We decided to search for available analogs of **16** and purchase them because **16** showed such promising nanomolar functional activity towards CB1. Our main goal of the exploration of analogs was to find more active and selective compounds for the CB1 receptor with higher polar surface area (to help limit CNS effects). We purchased five compounds which differed at the 5-hydroxyl group of the 1,5-dihydroxybenzene position of compound **16**, and labeled them **PCB**-**161** to **PCB**-**165** (Figure 9).

**Figure 10:**
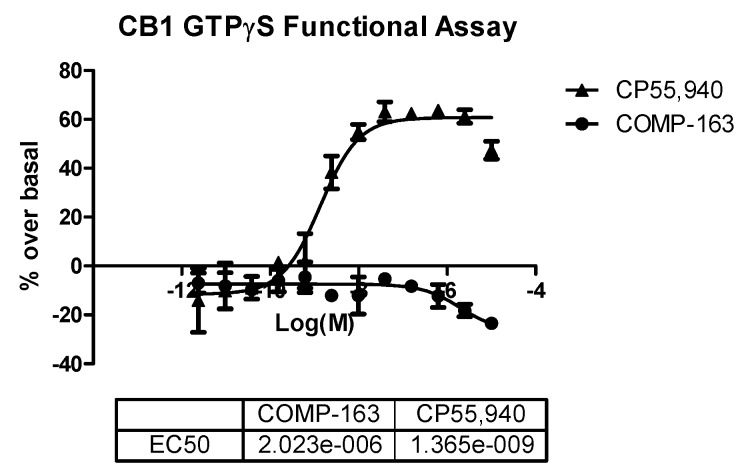
GTPγS functional curves for the CB1 receptor of compound **PCB-163** (abbreviated as **COMP-163**).

The benzyl (**PCB-161**) and *p*-bromobenzyl (**PCB-162**) substitutions at the 5-hydroxyl group on the 1,5-dihydroxybenzene of compound **16** resulted in compounds that possessed >10 μM CB activity (Table 2), which can therefore be considered as inactive. The *p*-fluorobenzyl substituted analog **PCB-163** was a potent and CB1-selective compound. Compound **PCB-163** was tested in the CB1 GTPγS functional assay and was found to be a CB1 inverse agonist with an EC_50_ of 2.023 μM (Figure 10).

**Figure 11:**
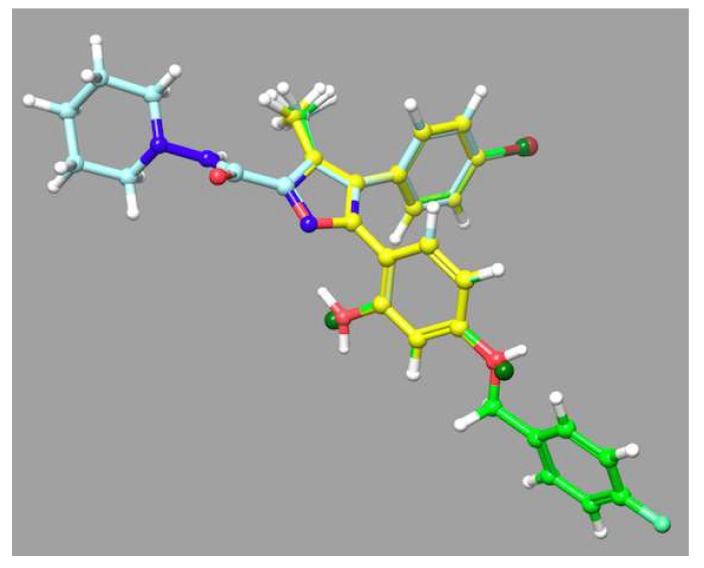
Overlay representation of compounds **16** (carbon in yellow) and **PCB-163** (carbon in green) with rimonabant (carbon in light blue).

Interestingly, the *n*-hexyl substitution at the 4-position and the methoxyl at the 5-position resulted in **PCB-164**, which had high affinity for CB2 and was a CB2-selective compound (K_i_ = 0.1556 ± 0.0173 μM) (Table 2). These experimental results confirmed for the analogs of **16** that for CB1 activity and selectivity, the 4-bromophenyl moiety is necessary along with the *p*-fluorobenzyl group (**PCB-163**), while for CB2 activity and selectivity, *n*-hexyl substitution at the 4-position and the methoxy at the 5-position (**PCB-164**) are critical. We aligned compounds **16**, **163**, and well-known inverse agonist rimonabant to see the structural similarity between them (Figure 11). The 3D structures of rimonabant and **PCB-16** and **PCB-163** show some similarity. The methylpyrazole core in rimonabant is replaced by a methylisoxazole ring in **PCB-16** and **PCB-163**. In addition, arms 1 and 2 of rimonabant (chlorophenyl and 1,3-dichlorophenyl groups) can serve as bioisosteres for the bromophenyl and 1,3-dihydroxyphenyl groups in **PCB-16**. The main differences between the structures is that arm 3 in rimonabant (piperidinyl carboxamide) is not present in **PCB**-**16** and **PCB-163**. Instead, the *p*-fluorobenzyl moiety is attached to the analogous arm 2 (**PCB-163**). Only the pyrazole-3 carboxamide and the *p*-fluorobenzyl moiety (**PCB**-**163**) do not align well with rimonabant; otherwise, the rest of the core structures matched well in their 3D arrangement (Figure 11). The shape similarity score of compound **16** with rimonabant was calculated to be 0.75.

## 3. Materials and Methods

### 3.1. Computational Methods and Approaches

#### 3.1.1. Protein Preparation, Receptor Grid Generation, and Amino Acid Numbering System

The 3D coordinates of our reported antagonist-bound CB1 receptor model [48] were used in the structure-based virtual screening of the ZINC12 database purchasable natural products subset with the goal of the identification of new natural product chemotypes as CB ligands. For protein–ligand interaction studies, a CB2 model [54] based on the bovine rhodopsin template was used. The protein was prepared using the Protein Preparation Wizard (PPW) [55] implemented in the Schrödinger suite that involved steps including addition of hydrogens, bond order, and atomic charge assignment, and optimization of the local positioning of all atoms including hydrogen. The maximum root-mean-square deviation (RMSD) of the atom displacement for terminating the minimization step was set to be less than 0.5 Å. One of the key residues, Trp279^5.43^ [56,57] located in TM5, was used as a centroid for receptor grid generation, and Lys192^3.28^ [58] located in TM3 was used as an H-bond constraint for the virtual screening study. The virtual screening workflow (VSW) of the Schrödinger suite was used for in silico screening of the prepared database.

The standard Ballesteros and Weinstein amino acid numbering system [59] (given in superscript) is used for referring to specific amino acids in the trans-membrane helices of the CB1 receptor. In general, the Ballesteros–Weinstein numbering was used to indicate the relative position of amino acid residues in the CB1 and CB2 receptor sequences including the TM helices.

#### 3.1.2. Database Preparation

A commercially available ZINC12 subset of 181,317 natural products downloaded from http://www.zinc.docking.org was prepared at physiological pH (7.4) to generate all of the possible tautomers and ionized states [60]. The prepared database with ~0.3 million compounds was filtered for drug-likeness using custom filtration criteria (MW ≤ 700 Daltons; LogP ≤ 5; No. of HBA ≤ 10; No. of HBD ≤ 5; No. of RB ≤ 10, and Total Polar Surface Area ≤ 140 Å^2^) that afforded a total of 278,037 compound structures.

#### 3.1.3. Structure-Based Virtual Screening: Docking and Scoring

The docking of the filtered set of 278,037 compounds into the generated CB1 receptor grid was accomplished in two steps. In the first step, the Glide standard precision (SP) method and flexible ligand sampling were used [50]. During docking, a reported key residue Lys192^3.28^ [58] was used as an H-bond constraint. In the second step, the top 2000 ranked compounds were then subjected to extra precision (XP) [51] docking into the generated CB1 receptor grid with an H-bond constraint on Lys192^3.28^ to eliminate false positives that may result from SP docking. The XP docking method applies a more extensive sampling and advanced scoring algorithm, and hence is computationally more intensive. The top ranked compounds from the XP docking were considered for further postprocessing, clustering, binding mode analysis, and final hit selection. The CB2 docking was done only for those molecules that were not selective in the CB1 and CB2 radioligand-binding assay. The CB2 receptor grid was prepared by choosing the centroid of residues Lys109^3.28^, Ser112^3.31^, Phe117^3.36^, Trp194^5.43^, Trp258^6.48^, Lys278, and Ser285^7.39^ as reported in our previous publication [29], and the docking was carried out with the same protocol as for the CB1 model. In the XP docking, no constraint was applied for CB2 receptor docking. The resulting docked poses were analyzed to understand the putative binding mode of ligands with the CB2 receptor. 

#### 3.1.4. Hit Postprocessing and Selection of Hits

A total of 618 compounds that resulted from the second step of XP docking were considered for further analysis. Considering a GlideScore cutoff of −8.00 kcal/mol, a total of 192 compounds were identified. These 192 compounds were then clustered using docking score and 2D fingerprint properties of ligands using the Canvas [61] module of the Schrödinger software suite (www.schrödinger.com). The final assessment of potential CB1 hits was done by visual inspection of the receptor–hit interaction geometry. In general, the visual inspection relied on (1) the formation of an H-bond interaction between the ligand and Lys192^3.28^ of the CB1 receptor; (2) favorable orientation of the hydrophobic part of the ligand into the receptor active site; and (3) hydrophobic contacts between ligand and receptor. Altogether, a set of 32 hits was identified, and from these, 18 structurally diverse compounds were selected because of their ease of purchasability (cost and immediate availability).

#### 3.1.5. Procurement, Purity Assessments, and Characterization of Selected Hits

All tested compounds were purchased from InterBioScreen Ltd., Chernogolovka, Russia [62]. The vendor verified that each compound had >92% purity by NMR (Bruker Avance 400 MHz, Billerica, MA, USA) and mass spectrometry (MS) (Waters ZQ^TM^, Milford, MA, USA). To further validate the purity of the purchased compounds, we performed characterization using HPLC (Waters Alliance HPLC, Milford, MA, USA), NMR (Bruker Avance 400 MHz), and MS (Waters ZQ^TM^) methods, and found >95% purity for each compound.

### 3.2. Biological Methods

#### 3.2.1. Reagents

CP55,940 was purchased from Tocris Bioscience (Minneapolis, MN, USA). BSA, Trizma^TM^ hydrochloride (Tris-HCl), penicillin and streptomycin, nonenzymatic cell dissociation solution, and guanosine 5′-diphosphate (GDP) were purchased from Sigma-Aldrich (St. Louis, MO, USA). [^3^H]-CP55,940 and MicroScint^TM^-20 were purchased from PerkinElmer (Waltham, MA, USA). Membrane preparation was made using a 50 mM Tris-HCl buffer with pH 7.4. Dilutions of membrane, radioligand and control/test compounds were made in a Tris-EDTA buffer (50 mM Tris-HCl, 20 mM EDTA, 154 mM NaCl, and 0.2% fatty-acid BSA), with pH = 7.4.

#### 3.2.2. Cell Culture

Human embryonic kidney 293 (HEK293) cells were purchased from ATCC, Manassas, VA. Cells were grown in 150 cm^2^ Corning culture dishes with Dulbecco’s modified Eagle’s medium (DMEM) and Ham’s F12 medium supplemented with 10% fetal bovine serum (FBS), 2 mM glutamine, penicillin (100 U/mL), and streptomycin (100 µg/mL) in an atmosphere of 5% CO_2_.

#### 3.2.3. Transfection and Stable Expression of CB1 and CB2 Receptors in Mammalian Cell Lines

HEK293 cells were collected and transiently transfected with full-length human recombinant cDNA (OriGene, Rockville, MD, USA) containing expression clones to generate separate cell lines expressing either the CB1 or CB2 receptors (50 µg/mL) using electroporation (70 ms, single pulse, 150 volts). Transfected cells were grown in a 150 cm^2^ Petri dish at 37 °C. G418 antibiotic solution (800 μg/mL) was used for selection. After selection, the HEK293 cells were further cultured until single colonies were obtained. The colonies with binding ratio (%) over 80% were chosen for binding and functional assays.

#### 3.2.4. Membrane Preparation

Cell plasma membranes were prepared from cells with stable expression of CB1 or CB2 receptors. Cells grown to confluency were lysed and scraped in cold 50 mM Tris-HCl buffer at pH 7.4 and then centrifuged at 1000 rpm for 10 min at 4 °C. The supernatant was discarded while the pellets were resuspended in the same buffer. A Sonic Dismembrator Model 100 (Fisher Scientific, Pittsburgh, PA, USA) was used to homogenize the cell suspension for 30 s, which was then centrifuged at 3165 rpm for 10 min at 4 °C to separate the membranes and cytosolic fractions. The supernatant was saved, and the pellet underwent the suspension and homogenization process repeated two more times with the same conditions. The supernatants were combined and centrifuged at 13,650 rpm for 40 min at 4 °C. The pellet was re-suspended in cold 50 mM Tris-HCl buffer, aliquoted into 2 mL vials, and stored at −80 °C. The total membrane protein concentration was measured using a Pierce BCA Protein Assay Kit (Thermo Scientific, Rockford, IL, USA) as per the manufacturer’s protocol.

#### 3.2.5. Radioligand Receptor Binding Studies

Competitive binding assays were performed with a modified rapid filtration assay described by Ma et al. (2007) [63] and Felder et al. (1992) [64]. Briefly, cell membranes (10 µg) were incubated with 0.5 nM [^3^H]-CP55,940 and test compounds in 50 mM Tris-EDTA buffer (50 mM Tris, pH 7.4, 20 mM disodium EDTA, 154 mM NaCl, and 0.2% bovine serum albumin) for 2 h at 37 °C with gentle shaking. Each test well contained 50 μL of radioligand ([^3^H]-CP55,940); 50 μL of compound, control, or vehicle; and 100 μL of cell membrane. Nonspecific binding was determined using 10 μM CP55,940 as a positive control and total binding was ascertained with 0.1% DMSO in Tris-EDTA buffer. The reaction was terminated via rapid vacuum filtration with cold Tris-HCl with 0.1% BSA through a 96-well UniFilter GF/C filter precoated with 0.5% Polyethyleneimine (PEI,) to separate bound and unbound radioligand. Filter plates were dried at 50 °C for at least 30 min, then 25 μL MicroScint-20 was applied to each filter and the plates were read on a TopCount NXT HTS Microplate Scintillation Counter (PerkinElmer, Waltham, MA). Filter bound radioactivity was recorded in counts per minute (CPM). Specific binding was defined as the difference between the binding that occurred in the presence and the absence of 1 μM unlabeled CP55,940.

The K_d_ of the radioligand (CP55,940) for each receptor (CB1 or CB2) was determined using membrane evaluation and saturation binding experiments. The membrane evaluation experiment was performed by incubating 1–10 μg of protein membrane with 1 nM [^3^H]-CP55,940. The percent binding of the nonlabeled control to the receptor was calculated using total, specific, and nonspecific binding. The optimal membrane concentration was decided on the basis of total binding with high signal counts and good percent binding (>90%) of CP55,940. The saturation assay involved incubation of optimal membrane concentration and 0–10 nM of [^3^H]-CP55,940 with 10 μM of a nonlabeled CP55,940 or 0.1% DMSO in buffer. All the experimental data were analyzed using a nonlinear regression curve fit model using GraphPad Prism 5.0 software (GraphPad Software, Inc., San Diego, CA, USA) and the K_d_ value was calculated. Each compound was tested in triplicate, unless stated otherwise.

Preliminary screening was performed at 10 μM using the optimal concentration of membrane with a radioligand concentration of ≤K_d_. Percent displacement of radioligand was determined using the following equation:

(1) $$\;\%\;displacement\;of\;radioligand=100\;–\left(\frac{compound.CPM-nonspecific.CPM}{specific.CPM\;}\times 100\right)\;$$

The IC_50_ and K_i_ values were calculated from a nonlinear regression curve fit model using GraphPad Prism 5.0 software (GraphPad Software, Inc., San Diego, CA, USA).

#### 3.2.6. CB1 GTPγS Functional Assay

The CB1 GTPγS functional assays were performed as previously described [65,66] with some modifications. The assays were performed in 250 mL of 50 mM Tris-HCl, pH = 7.4, 150 mM NaCl, 9 mM MgCl_2_, 0.2 mM EDTA, 1.4 mg/mL essentially fatty acid free BSA, 50 pM **[**^35^S]-GTP**γS** ([^35^S]-guanosine 5′-(γ-thio) triphosphate (PerkinElmer, Waltham, Massachusetts)), and 30 μg of protein per well harvested from HEK293 cells stably transfected with a plasmid overexpressing the human cannabinoid type 1 receptor. Agonist assays were run with 12 independent 4-fold serial dilutions in triplicate of the test compound and CP55,940 concentrations from 10 mM to 2.4 pM. Controls consisted of Emax (10 μM of unlabeled CP55,940), nonspecific binding (40 mM of nonlabeled GTPγS salt), and basal (vehicle only). Plates were incubated for 90 min at 37 °C with gentle agitation in 96-well microplates, harvested with a PerkinElmer FilterMate Harvester through Unifilter GF/B filter plates (prewetted for 30 min with 0.3% BSA), and then washed 10× with ~300 mL of ice-cold 50 mM TrisHCl, pH 7.4. The filter plates were dried at 50 °C for at least 30 min. The radioactivity retained on the filters was quantified by adding 50 mL MicroScint20 per well, incubating the filter plate overnight at room temperature to allow the radioactivity to solubilize into the scintillation fluid, and counting on a TopCount NXT Microplate Scintillation counter [65,67,68]. Percent over basal was calculated in Microsoft Excel by subtracting the mean basal control from each value obtained and then dividing by the basal specific activity (mean basal control∓mean nonspecific binding control). Dose response curves (± SEM) of percent over basal versus log of the molar concentration of unlabeled ligand(s) were generated by a nonlinear curve fit model using GraphPad Prism 5.0 software.

## 4. Conclusions

Through the in silico protein structure-based screening of the natural product subset of the ZINC12 database against a CB1 receptor model, we identified four small molecules as significant cannabinoid ligands. These compounds exhibited low micromolar or nanomolar displacement of the CB1 and CB2 receptor, and represent novel, natural product chemotypes which can be further optimized for improved affinity and selectivity toward one particular CB receptor. Notably, the most promising compounds **2**, **12**, and **16** were tested for functional phenotype (agonist/antagonist) in the GTPγS cannabinoid functional assay and found to be CB1 receptor inverse agonists. The inverse agonist nature of these compounds on CB1 receptors validated our CB1 model because these molecules were identified through the SBVS of the inactive state of the CB1 model. The identified hits exhibited strong interactions with both of the CB receptor subtypes, and their docking poses and scores explained well the observed binding affinity. Further, our structural exploration of analogs of identified hit **16** resulted in nanomolar range compounds which showed preference for CB1 (PCB-163) or CB2 (PCB-164). In the functional assay, PCB-163 was shown to be an inverse agonist of the CB1 receptor. PCB-163, which has a *p*-fluorobenzyl group (R_3_), showed high affinity and selectivity for the CB1 receptor. As per our docking analysis, we believe that the substitution of various electron-withdrawing groups (such as F, Cl, Br, and I) at different positions (*ortho*, *meta*, and *para*) of this moiety and testing similar substituents at the *X* position (cf. Figure 9) may lead to better hits. Docking studies showed that compound **16** formed similar interactions with the CB1 receptor to those found for rimonabant. Importantly, these scaffolds are structurally distinct from those of known CB1 inverse agonists. This work sets the stage for further research towards the development of novel CB1 inverse agonists through modification/optimization of molecular properties of the scaffolds such as the polar surface area and hydrophilicity, in order to be able to avoid the central activity observed with rimonabant.

## Figures and Tables

**Table 1:** The binding affinities (K_i_ and IC_50_) of selected compounds against CB1 and CB2 receptors.

Compound	IC_50_ ± SEM (μM)		K_i_ ± SEM (μM)	
	CB1	CB2	CB1	CB2
**2** *^a^*	3.2 ± 1.0	3.25 ± 0.5	1.62 ± 0.5	1.62 ± 0.51
**12** *^ab^*	>5.3 ± 1.4	>5.93 ± 0.8	>2.6 ± 0.7	>2.97 ± 0.4
**16**	20.8 ± 4.9	ND *^c^*	10.4 ± 2.5	ND *^c^*
**16** *^a^*	0.242 ± 0.07 *^d^*	ND *^c^*	0.121 ± 0.04 *^d^*	ND *^c^*
**18** *^e^*	24.6 ± 2.6	0.101 ± 0.035	12.3 ± 1.3	0.051 ± 0.01
CP55,940	9.84 nM	8.62 nM	4.9 nM	4.31 nM

^*a*^ Represents compound’s affinity up to its solubility limit; ^*b*^ racemic compounds; ^*c*^ ND = not determined because the percent displacement at 10 μM was low; ^*d*^ data from Figure 4, for which CP55,940 K_i_ = 1.267 ± 0.13 nM; ^*e*^ the chromophore nature of the compound may have interfered with the radioligand detection (cf. main text).

**Table 2:** The percent (%) displacements and the binding affinities (K_i_) of compound **16** analogs against CB1 and CB2 receptors in radioligand competition assays.

Compound	MW	(% Displacement at 10 µM)		K_i_ ± SEM (μM)	
		CB1	CB2	CB1	CB2
**PCB-161**	436.3	37.4	44.6	>10.0	>10.0
**PCB-162**	436.3	3.9	28.4	ND	>10.0
**PCB-163**	454.3	72.0	81.1	0.4870 ± 0.1516	1.084 ± 0.141
**PCB-164**	444.4	29.8	97.2	NC ^#^	0.1556 ± 0.0173
**PCB-165**	321.3	33.0	-	>10.0	-
CP55,940	376.6			0.002162 ± 0.000420	0.001185 ± 0.000128

^#^ “Not converged” means that the binding curve did not fit the expected sigmoidal shape.

## Supplementary Materials

The following are available online, Figure S1: Chemical structures of all 18 selected hits used for in vitro study; Figure S2: Binding curves for compounds **7**, **15**, and **17** obtained from the radioligand competitive binding assay; Table S1: Percentage displacement of radioligand at CB1 and CB2 receptors for all 18 screened compounds; Table S2: Physicochemical and other selected properties related to drug-likeness for all 18 tested hits.
