# Supplementary material for: Structure-Based Identification of Potent Natural Product Chemotypes as Cannabinoid Receptor 1 Inverse Agonists
Source: Molecules. 2018 Oct 13;23(10):2630. doi: 10.3390/molecules23102630 (PMC6222380; doi:10.3390/molecules23102630)
Supplement: Supplementary file 1 [file molecules-23-02630-s001.pdf]

Supporting Information

Structure-Based Identification of Potent Natural Product Chemotypes as Cannabinoid Receptor 1 Inverse Agonists

Pankaj Pandey [1], Kuldeep K. Roy [1,3], Haining Liu [1], Guoyi Ma [1], Sara Pettaway [1,§], Walid F. Alsharif [1,⊥], Rama S. Gadepalli [1], John M. Rimoldi [1,2], Christopher R. McCurdy [1,2,#], Stephen J. Cutler [1,2,†] and Robert J. Doerksen [1,2,*]

[1] Department of BioMolecular Sciences, Division of Medicinal Chemistry, School of Pharmacy, The University of Mississippi, University, MS 38677, USA
[2] National Center for Natural Products Research, School of Pharmacy, The University of Mississippi, University, MS 38677, USA
[3] National Institute of Pharmaceutical Education and Research, 168, Manicktala Main Road, Kolkata 700054, WB, India
[*] Correspondence: rjd@olemiss.edu; Tel.: +1-662-915-7052

Footnote: Current addresses of co-authors;

[§] Eshelman School of Pharmacy, University of North Carolina, Chapel Hill, NC 27599, USA

[⊥] Department of Psychiatry and Behavioral Neurosciences, Wayne State University, School of Medicine, Detroit MI 48202, USA

[#] Department of Medicinal Chemistry, College of Pharmacy, University of Florida, Gainesville FL 32610, USA

[†] College of Pharmacy, University of South Carolina, Columbia SC 29208, USA

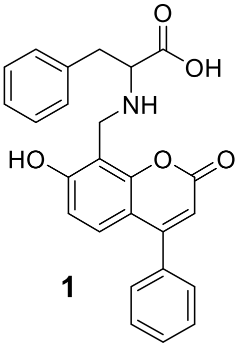

1

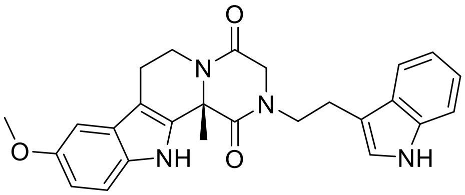

2

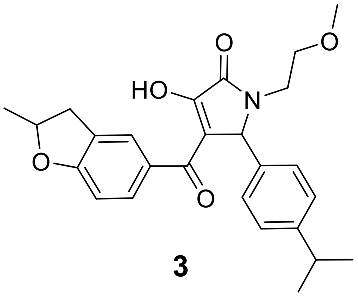

3

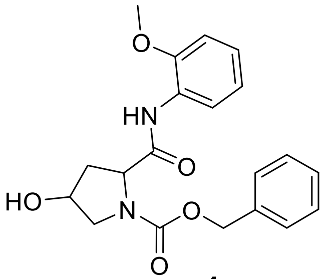

4

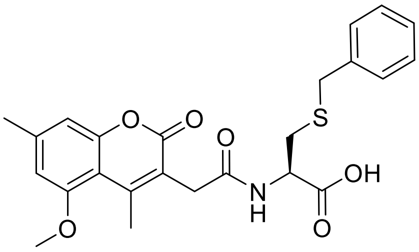

5

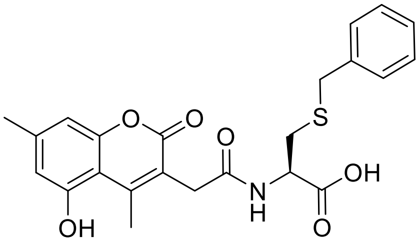

6

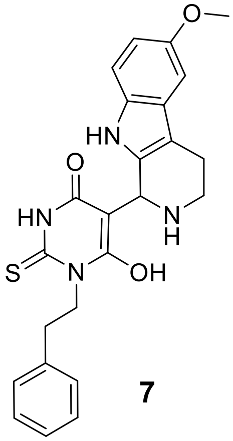

7

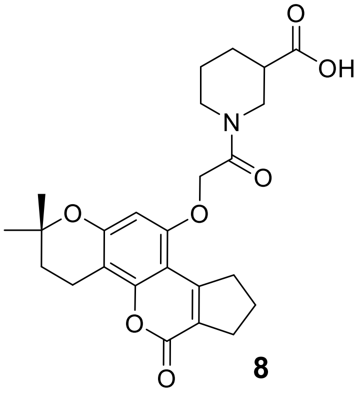

8

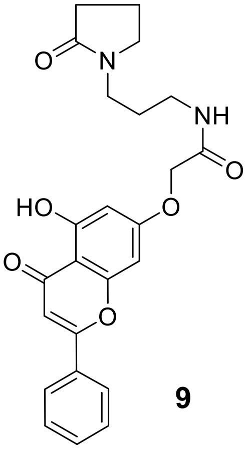

9

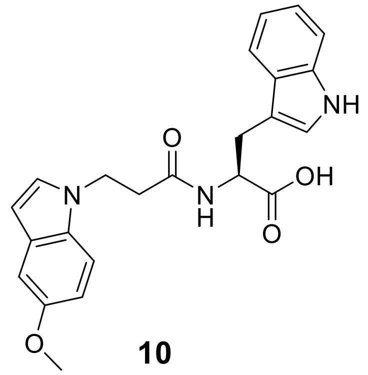

10

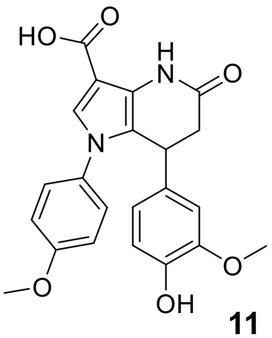

11

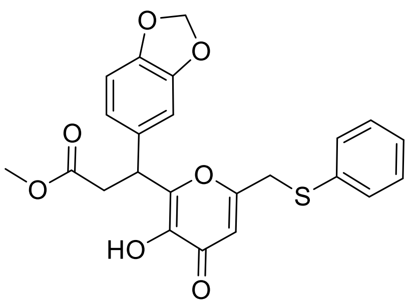

12

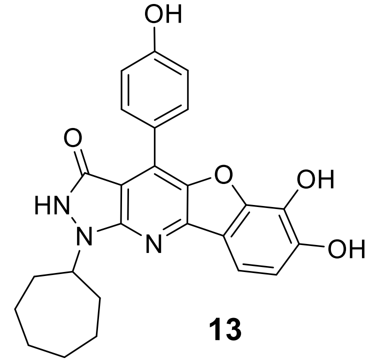

13

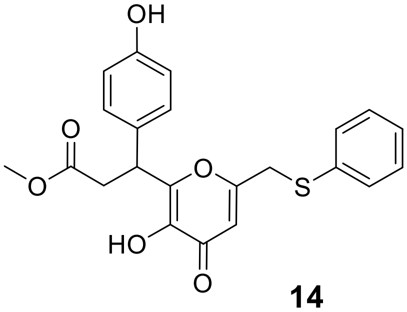

14

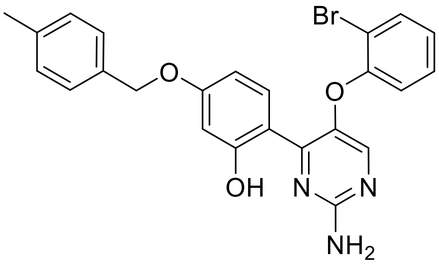

15

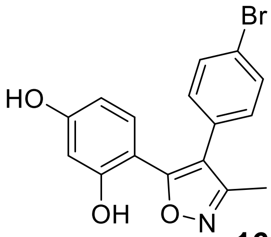

16

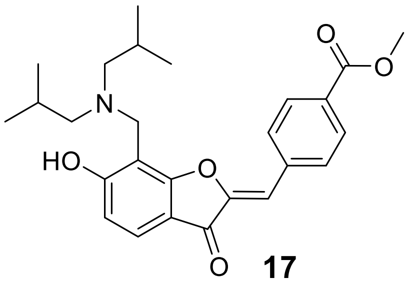

17

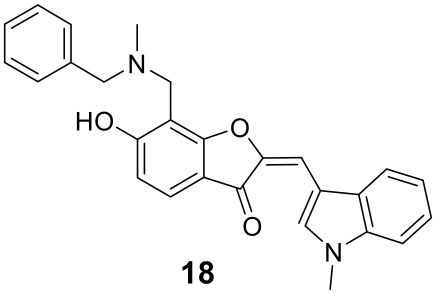

18

Figure S1. Chemical structures of the eighteen selected hits used for *in vitro* study.

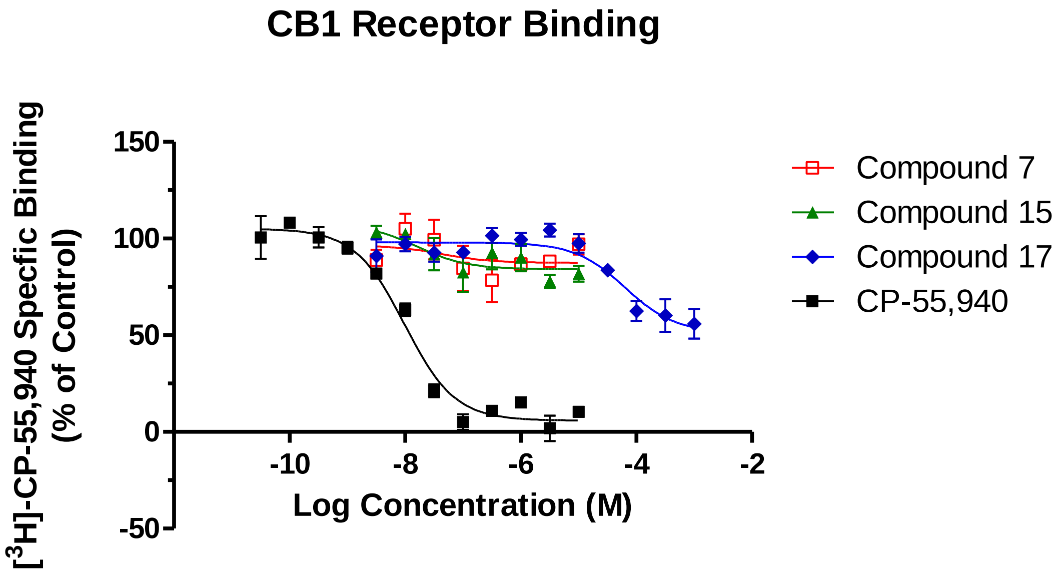

Figure S2. The displacement binding curves for compounds **7**, **15** and **17** obtained from the radioligand competitive binding assay. CP55,940 was used as a positive control.

Table S1. The percentage (%) displacement of radioligand at CB1 and CB2 receptors for all eighteen screened compounds.

Compound number	Sample name	ZINC code	Docking score ‡	% displacement at 10 μM	
				CB1	CB2
**1***	STOCK1N-06857	ZINC00940539	−10.153	14.1	-
**2**	STOCK1N-34850	ZINC09033729	−9.975	81.8	67.2
**3***	STOCK1N-43108	ZINC09374751	−9.74	-	51
**4***	STOCK1N-43876	ZINC00861983	−9.60	13.9	-
**5**	STOCK1N-51636	ZINC02118939	−11.119	14	15.5
**6**	STOCK1N-53876	ZINC02123699	−11.517	28	26.7
**7***	STOCK1N-62719	ZINC20755817	−12.295	49.7	15.7
**8***	STOCK1N-68672	ZINC08879802	−9.777	30.5	7
**9**	STOCK1N-69353	ZINC12896219	−9.804	28.6	30.2
**10**	STOCK1N-73177	ZINC20763677	−9.014	30.2	17.3
**11***	STOCK1N-75513	ZINC40313209	−10.73	37.6	13.1
**12***	STOCK1N-79387	ZINC85875400	−11.723	69.9	63
**13**	STOCK1N-79693	ZINC85877048	−9.638	23.1	37.4
**14***	STOCK1N-80140	ZINC85879663	−9.967	18.7	30
**15**	STOCK5S-41625	ZINC02229040	−10.459	57.6	35.5
**16**	STOCK6S-13543	ZINC06757795	−9.378	61.8	40.2
**17**	STOCK6S-34022	ZINC08823658	−9.818	50.4	35
**18**	STOCK6S-39252	ZINC28182437	−10.436	69	61.2

‡ Kcal/mol; * Racemic

Table S2. Physicochemical and other selected properties related to drug-likeness for all eighteen tested hits.

Compound number	MW	QPLogP	#HBA [a]	#HBD [b]	PSA [c]	#RB [d]
**1** [e]	415.45	1.710	7	3	117.402	8
**2**	442.52	3.886	6	1	87.903	4
**3** [e]	435.519	4.406	7.2	0	91.620	7
**4** [e]	370.404	3.233	7.45	2	98.812	6
**5**	455.525	4.160	7.5	1.25	123.966	9
**6**	441.498	3.403	7.5	2.25	137.364	9
**7** [e]	448.539	4.696	4.25	3	103.898	5
**8** [e]	455.507	2.872	9	1	126.134	4
**9**	436.463	2.170	8	1	132.713	8
**10**	405.45	4.572	4.5	2.25	107.384	8
**11** [e]	408.410	3.121	5.75	2	129.267	4
**12** [e]	440.47	3.924	7.25	1	109.649	8
**13**	445.474	3.789	4.75	4	123.602	5
**14** [e]	412.456	3.350	6.5	2	113.379	9
**15**	478.344	4.918	4.5	3	83.400	8
**16**	346.18	3.153	3	2	69.421	2
**17**	437.535	3.971	7.25	1	94.545	10
**18**	424.5	4.597	5.25	1	65.049	7

[a] Number of Hydrogen Bond Acceptors; [b] Number of Hydrogen Bond Donor; [c] Polar Surface Area; [d] Number of Rotatable Bonds; [e] Racemic.
